# Changes in Core Temperature of Cyan-Shank Partridge Chickens Exposed to Continuously Increased Ambient Temperature at Different Relative Humidity Levels

**DOI:** 10.3390/ani15060820

**Published:** 2025-03-13

**Authors:** Chen Wang, Ketian Chen, Haocong Xu, Le Liu, Longshen Liu, Chunmei Li, Yansen Li

**Affiliations:** 1 College of Animal Science and Technology, Nanjing Agricultural University, Nanjing 210095, China; 2024205036@stu.njau.edu.cn (C.W.); ketianchen@stu.njau.edu.cn (K.C.); 2022805130@stu.njau.edu.cn (H.X.); lle@stu.njau.edu.cn (L.L.); chunmeili@njau.edu.cn (C.L.); 2 College of Artificial Intelligence, Nanjing Agricultural University, Nanjing 210095, China; liulongshen@njau.edu.cn

**Keywords:** Cyan-shank partridge chickens, relative humidity, body core temperature, inflection point temperature, ages

## Abstract

(1) Background: This study aimed to investigate the changes in core temperature of Cyan-shank partridge chickens with continuously increased ambient temperature at different relative humidity (RH) levels. (2) Methods: Thirty birds were selected at the age of 35 days and randomly divided and housed in three artificial climate chambers. Each chamber was set at one of three different RH levels (50%, 65%, and 80%), and the ambient temperature was increased by 1.0 °C per 0.5 h from 24.0 to 34.0 °C. The core temperature was tested at 1.0 h intervals, and the ambient temperature in the chambers was recorded using mini temperature data loggers. Data were collected continuously for three days at the ages of 35, 42, and 49 days. The broken-line model (BLM) was used to calculate the inflection point temperature (IPT) and basal core temperature (constant). (3) Results: Both RH and age had no significant influence on the IPT. With increasing ambient temperature, the average IPT values of birds measured at the three ages were 26.52, 27.02, and 26.71 °C at the RH levels of 50%, 65%, and 80%, respectively. A significant downward trend in basal core temperature was observed as the birds aged from 35 to 42 days (*p* < 0.05). (4) Conclusions: the core temperature of the chicken gradually decreased as the age increased. During the hot summer months, it is better to keep the ambient temperature less than 26.5 °C to avoid the excessive increase in core temperature in Cyan-shank partridge chicken at the ages from 35 to 49 days.

## Simple Summary

This study explores the real-time variation of core temperature in Cyan-shank partridge chickens with continuously increased ambient temperature at different relative humidity levels. The turning point of ambient temperature when core body temperature sharply increases was calculated. The results showed that the turning point of ambient temperature was in the range from 26.52 to 27.02 °C and basal core temperature decreased gradually as bird age increased from 35 to 49 days. These findings provide a data basis for attenuating heat stress-induced low efficiency of production in Cyan-shank partridge chickens.

## 1. Introduction

Poultry are susceptible to high ambient temperature due to their feather covering and lack of sweat glands [1]. As an important indicator in the poultry production process, ambient temperature plays a vital role in the growth and health of poultry [2,3,4]. The Cyan-shank partridge chicken has the physical characteristics of cyan-shanked feet; a thin head and feet; and back feathers in the colors of yellow, brown, or a blend of both [5]. This chicken is highly favored by local consumers due to its strong adaptability, rapid growth, excellent meat quality, and flavor [6]. However, the optimal settings of ambient temperature for the Cyan-shank partridge chicken remain unclear, resulting in frequent heat stress during the summer. As a valuable broiler breed, the Cyan-shank partridge chicken is native to China and mainly distributed in the Yangtze River basin and southern China [7]. Although the native chicken breed has good capacity of thermal regulation, it is still susceptible to heat stress in a high-temperature environment. Chicken houses are usually controlled at 18–25 °C and the RH is 45–55% [7,8]. However, the house ambient temperature always exceeds 25 °C and RH rises to the level above 80% during hot summer months in the area of south China [9,10]. The thermal comfort zone of chickens will vary depending on the real-time variation of RH [11]. Therefore, it is necessary to estimate the optimal ambient temperature within the chicken house.

The thermoneutral zone reflects the range of ambient temperature at which internal temperature regulation is solely achieved by controlling dry heat loss, indicating that the metabolic rate remains relatively constant without regulatory changes in heat production or evaporative heat loss [12,13]. The upper critical temperature of the thermoneutral zone can be calculated by analyzing the inflection point temperature (IPT) of the increases in evaporative heat loss or the increases in metabolic rate using the nonlinear segmental regression method. This method is also known as the broken-line regression, which always use the broken-line model (BLM) to calculate the IPT according to previous study [14,15]. Thus, the IPT of the increases in evaporative heat loss was defined as the evaporative upper critical temperature while the IPT of the increases in metabolic rate was defined as the metabolic upper critical temperature [13,16]. The core temperature refers to the temperature of the core part of the animal’s body, which can accurately reflect the internal temperature state of the animal. The IPT is the certain ambient temperature and is also noted as the basal core temperature, above which the body temperature of poultry starts to rise. A previous study found the trend of chicken core temperature was fitted to the BLM as ambient temperature increased and subsequently estimated the IPT of the increases in chicken core temperature using nonlinear segmental regression [17].

Poultry core temperature remains stable in the thermoneutral zone, but it is not possible to maintain a constant core temperature as the ambient temperature continuously increases. In addition, poultry core temperature was higher at a relative humidity (RH) of 85% than at RH levels of 35% and 50% [18]. This was because the poultry lack sweat glands for evaporative heat loss and only rely on the exposed skin of the head and legs to dissipate heat [19]. Previous studies reported that the upper critical temperature of the thermoneutral zone decreased with increasing age and body weight in broilers [20], suggesting that broilers had a different thermoneutral zone at different growth stages.

In order to improve their rearing efficiency, the rearing mode of Cyan-shank partridge chickens has gradually shifted to a multi-layer cage system in commercial farm settings [7,8]. Moreover, the body weight of the chicken surpassed 1 kg at the age of 35 days, coinciding with a higher growth rate. Thus, they became more susceptible to elevated ambient temperatures and faced increased risk of heat stress. Taken together, the present study focused on cage-reared Cyan-shank partridge chickens and measured real-time variation in the core temperature of birds at the ages of 35, 42 and 49 days exposed to continuously increased ambient temperature at different RH levels. The BLM of segmental regression was used to calculate bird IPT and constant core temperature as birds were exposed to a continuous increase in air temperature. The effect of RH and age on bird IPT, the slope, and the constant were tested to evaluate the thermal comfort zone of Cyan-shank partridge chicken at different ages or RH levels.

## 2. Materials and Methods

### 2.1. Birds and Treatments

**Figure 1:**
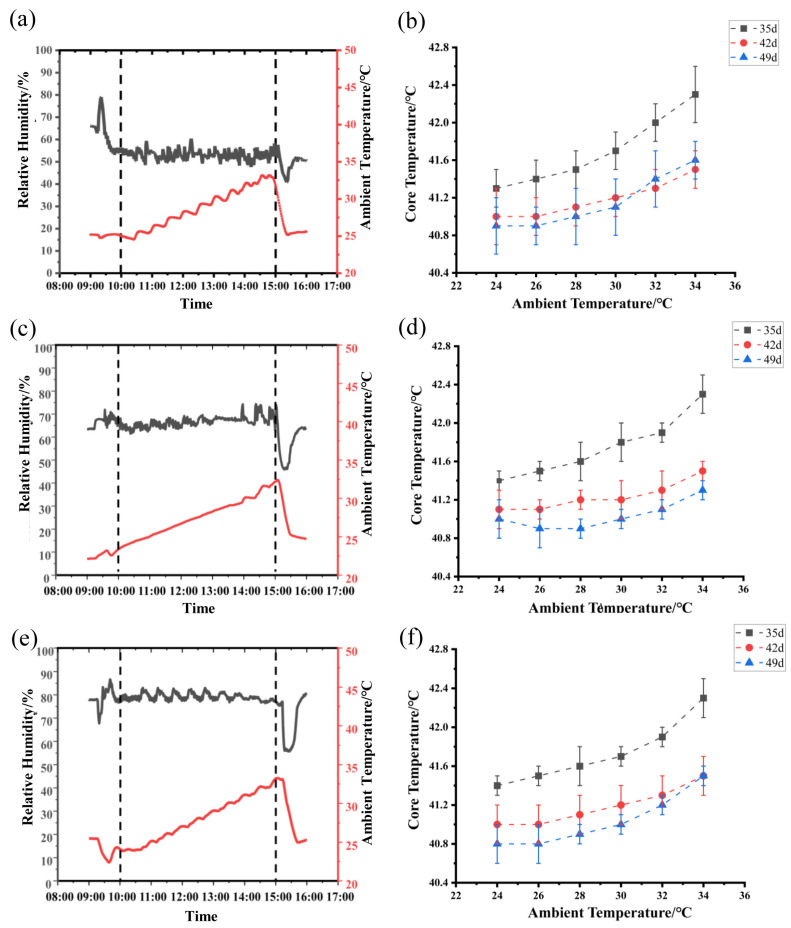
Real-time variations of ambient temperature and relative humidity in the controlled climate chambers and the changes in bird core temperature with increasing ambient temperature: (**a**) measured ambient temperature and relative humidity at 50% RH level, (**b**) changes in averaged core temperature at 50% RH level (*n* = 10), (**c**) measured ambient temperature and relative humidity at 65% RH level, (**d**) changes in averaged core temperature at 65% RH level (*n* = 10), (**e**) measured ambient temperature and relative humidity at 80% RH level, and (**f**) changes in averaged core temperature at 80% RH level (*n* = 10). The black solid lines represent the variations of RH and the red solid lines represent the changes of the ambient temperature in Figures (**a**,**c**,**e**).

This study was conducted in the artificial climate chambers (4.37 m L × 3.76 m W × 3.60 m H) at Nanjing Agricultural University. Thirty Cyan-shank partridge chickens at the age of 35 days with similar body weights (1094 ± 50 g, *n* = 30) were randomly divided into three groups and housed in three separate environmental chambers. Birds were raised in 3-layer steel cages, which had the following dimensions: 60 cm length, 30 cm width, and 35 cm height. One cage can hold two chickens at the density of about 12 birds per m^2^ according to the commercial farm setting [7]. Ten experimental chickens in the same treatment were fitted with leg bands, placed in ten cages, and raised together with the other non-experimental chickens in the cages. Each chamber was set at one of three different RH levels (50%, 65%, and 80%), and the ambient temperature was increased by 1.0 °C per 0.5 h from 24.0 to 34.0 °C. The RH settings were based on three typical RH levels that occurred in actual production in this region [7,8,9]. The humidity levels were set at 50% for optimal comfort, 65% to represent moderate conditions, and 80% to reflect the high humidity typically encountered in summer months. The wind speed inside the artificial climate chamber was around 0.2 m/s, which was at a level where the wind-chill effect was not significant [21]. The ambient temperature of the chamber was regulated by a PTC electric heater (DPF–1.3C, Han Yin Hardware Co., Luohe, China) and the heated air was sent into the chamber through pipes for circulation. The ambient temperature of each chamber increased from 24 °C at 10:00 a.m. to 34 °C at 15:00 p.m. The increased ambient temperature up-regulated the water-holding capacity of the chamber air, thereby causing a decrease in chamber RH. The chambers used a humidifier (LDR 0.035–0.7, Shang Yi Thermal Equipment Co., Zhangjiagang, China) to keep the RH at the same level by the evaporative humidification method. Thus, the real-time RH levels of the three chambers were measured (Table S2) and fluctuated around 50% (Figure 1a), 65% (Figure 1c) or 80% (Figure 1e). Diets were formulated to meet poultry requirements [22], and all birds were provided with ad libitum access to feed and water. The diet formulated for the chicken is presented in the Table S1. All experimental protocols were approved by the Institutional Animal Care and Use Committee of Nanjing Agricultural University (Certification No. SYXK2017-0007).

### 2.2. Data Measurements

The IPT of core body temperature was used as an indicator of mild heat stress to reflect the failure of sensible heat dissipation in chickens while the IPT of core body temperature was utilized as an indicator of severe heat stress to indicate that both sensible and latent heat dissipation were insufficient [23]. Thus, core body temperature was adopted to calculate the upper critical temperature of the thermoneutral zone by measuring the rectal temperature. With the increase in ambient temperature, the birds’ rectal temperature was measured six times at 1.0 h intervals and obtained from 24 °C at 10:00 a.m. to 34 °C at 15:00 p.m. using a rectal thermometer (TH-5, Physitemp, Clifton, NJ, USA). In order to reduce handling-induced change in core temperature, the side-pinning way was used to measure birds’ rectal temperature following capture from cages according to a previous study [24]. Rectal temperature measurements were conducted with the assistance of two individuals. One person was responsible for holding the bird, while the other performed the measurement using a thermometer specifically designed for small animals. The thermometer probe was inserted 2–3 cm into the rectum according to a previous study [25]. Two seconds later, the data displayed on the thermometer were recorded. The measuring system had an accuracy of ±0.1 °C. Additionally, ambient temperature and RH were recorded using a precision temperature and humidity meter (RC-4HC, Jiangsu Jingchuang Electric, Xuzhou, China). Data were collected continuously for three days over three time-intervals: from 34 to 36 days of age, from 41 to 43 days of age, and from 48 to 50 d of age. These three intervals were designated as representing 35 days of age, 42 days of age, and 49 days of age, respectively.

### 2.3. Statistical Analysis

Statistical analysis was conducted using SPSS 20.0 software and segmental regression analysis was used to calculate the IPT of the core temperature variations for each bird exposed to the continuous increase in ambient temperature. The parameter used in the BLM of each bird was the average based on 3 d trials. The initial values of all iterative parameters were uniformly set to 1. For the IPT parameter, its constraint range was defined within the interval from 24 to 34. The BLM of segmental regression was used according to a previous study [8], and is shown as follows:

When AT ≥ IPT, CT = C + Z × (AT − IPT);

When AT < IPT, CT = C.

where AT is the chamber ambient temperature (24 to 34 °C), C is a constant (basal core temperature), IPT is the inflection point temperature, CT is the response variable (real-time core temperature), and Z is the slope of the line (the change in Y with respect to the change in ambient temperature).

The IPT of each bird was calculated by averaging the bird IPTs measured during the 3 d trials. For birds at each RH level or age, the IPT was calculated by averaging the IPT of all birds reared in the same chamber. The normality of the data was verified using the Shapiro–Wilk test, and the homogeneity of variance was confirmed through the Levene variance homogeneity test. Subsequently, two-way ANOVA was carried out to examine the effect of age and RH on the parameters in the BLM of bird core temperature using a GLM procedure with SPSS 20.0 software. The data are expressed as “mean ± standard deviation”. To ascertain the significance of differences between means, Duncan’s post hoc test was applied, and statistical significance was established at *p* < 0.05.

## 3. Results

### 3.1. Changes in Chamber AT and Bird Core Temperature as Exposed to Different RH Levels

As illustrated in Figure 1, with the increasing ambient temperature in each chamber, the core temperature of birds in each chamber showed an elevated trend under three different RH levels. Moreover, birds had an obviously higher core temperature at the age of 35 days than those at the ages of 42 and 49 days under three different RH levels (Figure 1b,d,f) (Table S2). The trend of the core temperature variations was fitted to the BLM at different RH levels of 50% (Figure 1b), 65% (Figure 1d), and 80% (Figure 1f).

### 3.2. Estimating IPT Based on the Core Temperature Variations of Birds Exposed to the 50% RH Level

**Figure 2:**
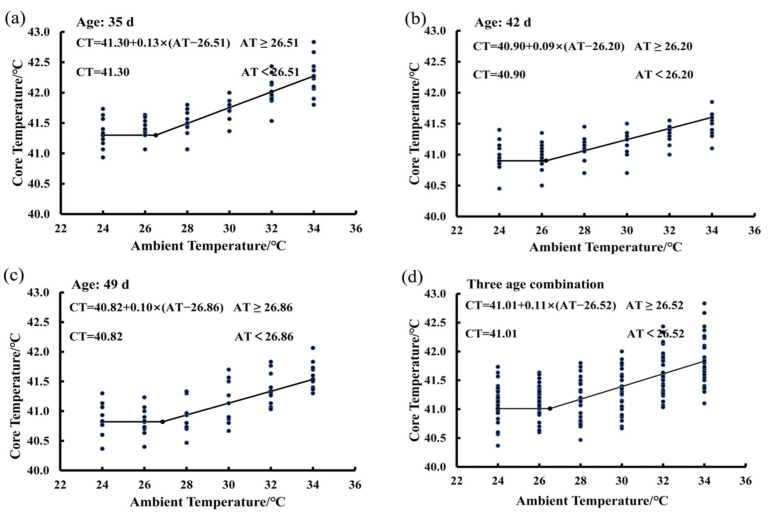
Estimating IPT based on the core temperature variations of birds exposed to the 50% RH level: (**a**) 35 days of age; (**b**) 42 days of age; (**c**) 49 days of age; (**d**) combination of the three ages.

In Figure 2, there are four functions showing the average BLM for birds at the ages of 35 days (Figure 2a), 42 days (Figure 2b), and 49 days (Figure 2c), as well as the average BLM for a combination of the three ages (Figure 2d). The average IPT of birds measured at three ages was 26.52 °C at 50% RH (Figure 2d). When the ambient temperature was lower than the IPT, the bird core temperature was a constant and the basal core temperature was 41.01 °C. When ambient temperature was higher than the value of 26.52 °C, bird core temperature increased in a linear manner by an average of 0.11 °C per degree Celsius increase in ambient temperature. The average IPT values were 26.51, 26.20, and 26.86 °C for birds at the ages of 35, 42, and 49, respectively (Figure 2a–c). The basal core temperature of birds decreased gradually with increasing age. The basal core temperature was 41.30 °C at the age of 35 days (Figure 2a), 40.9 °C at the age of 42 days (Figure 2b), and 40.82 °C at the age of 49 days (Figure 2c).

### 3.3. Estimating IPT Based on the Core Temperature Variations of Birds Exposed to the 65% RH Level

**Figure 3:**
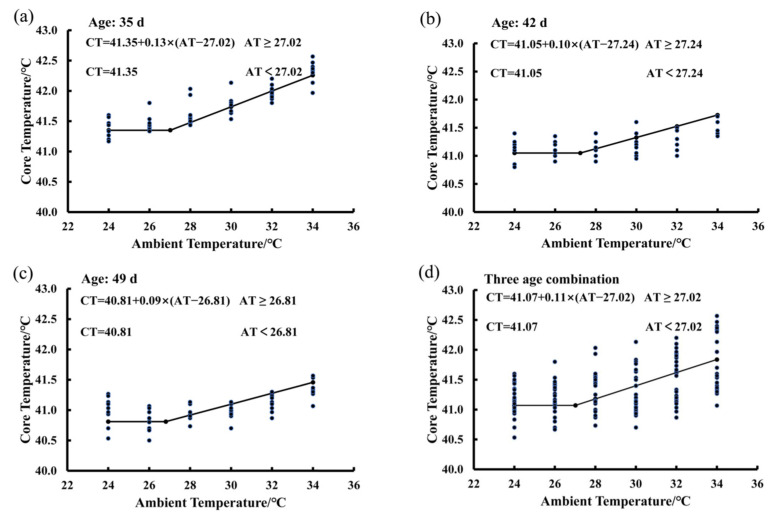
Estimating IPT based on the core temperature variations of birds exposed to the 65% RH level: (**a**) 35 days of age; (**b**) 42 days of age; (**c**) 49 days of age; (**d**) combination of the three ages.

In Figure 3, there are four functions showing the average BLM for birds at the ages of 35 days (Figure 3a), 42 days (Figure 3b), and 49 days (Figure 3c), as well as the average BLM for combination of the three ages (Figure 3d). The average IPT was 27.02 °C for birds at the ages from 35 to 49 days exposed to the 65% RH level (Figure 3d). When ambient temperature was lower than the IPT, the bird core temperature was a constant and the basal core temperature was 41.07 °C. When ambient temperature was higher than the value of 27.02 °C, bird core temperature increased in a linear manner by an average of 0.11 °C per degree Celsius increase in ambient temperature (Figure 3d). The average IPT values of birds were 27.02, 27.24, and 26.81 °C at the ages of 35, 42, and 49, respectively (Figure 3a,b). The basal core temperature of birds decreased gradually from 41.35 °C to 40.81 °C as the bird age increased from 35 to 49 days (Figure 3a,b).

### 3.4. Estimating IPT Based on the Core Temperature Variations of Birds Exposed to the 80% RH Level

**Figure 4:**
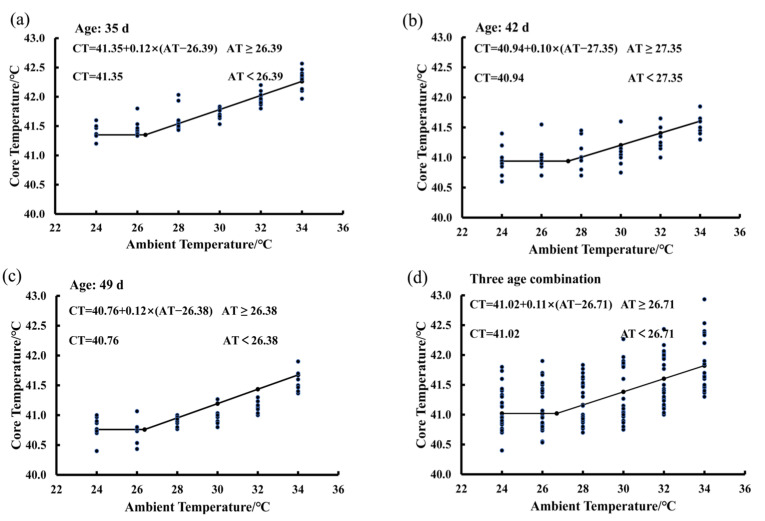
Estimating IPT based on the core temperature variations of birds exposed to the 80% RH level: (**a**) 35 days of age; (**b**) 42 days of age; (**c**) 49 days of age; (**d**) combination of the three ages.

In Figure 4, there are four functions showing the average BLM for birds at the ages of 35 days (Figure 4a), 42 days (Figure 4b), and 49 days (Figure 4c), as well as the average BLM for combination of the three ages (Figure 4d). The average IPT was 26.71 °C for birds at the ages from 35 to 49 days exposed to the 65% RH level (Figure 4d). When ambient temperature was lower than the value of 26.71 °C, the bird core temperature was a constant and the basal core temperature was 41.02 °C. When ambient temperature was higher than the value of 26.71 °C, bird core temperature increased in a linear manner by an average of 0.11 °C per degree Celsius increase in ambient temperature. The average IPT values were 26.39, 27.35, and 26.38 °C for birds at the ages of 35, 42, and 49, respectively (Figure 4a–c). The basal core temperature of birds decreased gradually from 41.35 °C to 40.76 °C as the bird age increased from 35 to 49 days (Figure 4a–c).

### 3.5. Effect of RH and Age on the IPT of Birds Exposed to the Increasing Ambient Temperature

As shown in Table 1, no significant difference was observed in the IPT based on two-way analysis. Both the constant and the slope decreased significantly as the bird age increased (*p* < 0.001). The constant of birds at the age of 42 days was lower than that of birds at the age of 35 days (*p* < 0.05) and higher than that of birds at the age of 49 days (*p* < 0.05). Birds had a higher slope at the age of 35 days than those at ages of 42 and 49 days (*p* < 0.05). Relative humidity had no significant influence on the IPT (*p* = 0.560), slope (*p* = 0.672), or constant (*p* = 0.430). No significant interaction of RH and age was observed in the IPT (*p* = 0.659), slope (*p* = 0.150), or constant (*p* = 0.728).

## 4. Discussion

The present study firstly detected the IPT of Cyan-shank partridge chicken at the RH levels of 50%, 65%, and 80% at the ages of 35, 42, and 49 days. With the increasing ambient temperature, the average IPT values of birds measured at the three ages were 26.52, 27.02, and 26.71 °C at the RH levels of 50%, 65%, and 80%, respectively. Both RH and age had no significant influence on the IPT, implying that this native chicken breed has a good thermal adaptability to continuously increased ambient temperature.

### 4.1. Changes in Core Temperature and IPT as Exposed to the Increasing Ambient Temperature

As the ambient temperature rose, birds increased heat dissipation and decreased heat production to maintain body core temperature [26]. A previous study reported hyperbaric oxygen increased body core temperature, heart rate, and body surface temperature when chickens rapidly increased peripheral blood circulation [27]. In White Leghorn laying hens aged 252 d, the rectal temperature did not change significantly when exposed to the ambient temperature lower than that of 30 °C, while the rectal temperature rose sharply when the ambient temperature exceeded 30 °C [28]. Moreover, in 35-week-old Jinghong laying hens, the estimated IPT of BLM ranged from 24.45 to 25.46 when they were exposed to increasing ambient temperature [17]. In addition to the differences in statistical methods, the remarkable difference in the IPT between two laying hen breeds might result from the difference in genetic background. These studies implied that there was a turning point of ambient temperature for birds when bird core temperature rose sharply with increasing temperature. In this study, the average IPT of birds measured at three ages was in the range from 26.52 °C to 27.02 °C at the RH levels from 50% to 80%. Thus, it is better to keep the ambient temperature less than 26.5 °C to maintain the basal core temperature of Cyan-shank partridge chicken at the ages from 35 to 49 days. Compared with younger chickens studied in the present study, adult Leghorn laying hens had a higher thermoregulatory turning point [28]. This might be ascribed to the fact that adult laying hens had larger combs and wattles, and a more streamlined body structure, and the Leghorn breed was noted for its relatively high activity levels [29]. Another reason might be the different bird density, which can affect heat dissipation. These features collectively affected the hens’ ability to dissipate heat, thus allowing them to maintain a higher turning point of body temperature when exposed to a high ambient temperature.

### 4.2. Changes in Slope in BLM with the Increasing Ambient Temperature and Age

In a commercial farm setting, the body weight of fast-growing broilers increases at a rapid rate with age [30]. The body temperature of broilers (Ross × Ross 718) increased progressively from 41.04 to 42.21 °C with age from 19 to 47 days in growing modern meat-type chickens [31]. In the present study, the basal core temperature of birds decreased gradually from 41.33 to 40.76 °C as the bird age increased from 35 to 49 days. This phenomenon may be explained by two possible reasons. Firstly, studies have shown that native chickens generally have better thermal regulation than commercial broilers, which is related to the different expressions of heat shock protein and cytokine genes [32,33]. Commercial broilers are bred through selective breeding to optimize growth rates and feed conversion rates. However, this selective breeding process may have weakened their thermal regulation. This might be because wild-type chickens exhibited higher levels of creatine kinase, lactate dehydrogenase and alanine aminotransferase than Arbor Acres (AA) chickens under heat stress [34]. This study also conclusively demonstrated that the gene encoding the thyroid-stimulating hormone receptor played a pivotal role in enhancing heat tolerance and facilitating adaptation to elevated ambient temperatures. In contrast, native chickens retained a better capacity for thermal regulation because they have not undergone similar high-intensity selection. Secondly, early-age heat exposure effectively reduces the body temperature in broilers under acute heat treatment [35]. The slope can be used as a marker to identify how quickly a chicken’s body temperature increases with the increasing ambient temperature. A previous study reported that the IPT was 27.82 °C and the slope of BLM ranged from 0.24 to 0.26 in AA chickens at the age of 21 days when exposed to an increasing ambient temperature [20]. In 35-week-old Jinghong laying hens, the slope of BLM ranged from 0.10 to 0.11 when exposed to an increasing ambient temperature [17]. In this study, the slope of BLM ranged from 0.09 to 0.13, which was higher than that of AA chicken, indicating Cyan-shank partridge chickens had a lower rate of increase in core temperature than AA chickens. The slope decreased significantly from 0.13 to 0.10 with the gradual increase in age, implying the increasing capacity of thermal regulation during the chicken’s growth. Taken together, the decreased basal core temperature with age might be attributed to good heat regulation.

### 4.3. Changes in the Parameters in BLM as Exposed to the Increasing RH

Air humidity affects the heat dissipation of chickens and their thermoregulation. Under a high ambient temperature, chickens primarily rely on rapid breathing to dissipate heat via evaporative cooling. When air RH is low, more moisture can evaporate from the respiratory system of chickens, making panting more effective in cooling the body. When the air RH increases, it is harder for air to absorb more moisture, causing a reduction in evaporative cooling and an increased body temperature. A previous study reported that RH significantly affected the chicken IPT of the skin surface temperature, and the IPT was higher at 50% RH than that at 85% RH [17]. However, this report observed that air RH had no significant effect on the IPT of the body core temperature, implying that high RH adversely affected skin heat dissipation and caused the inflection point of increased skin temperature to occur earlier when exposed to increasing ambient temperature. As a compensatory method, birds might elevate their skin surface temperature at a lower ambient temperature to enhance the dry heat dissipation. In this study, RH also had no significant effect on the IPT, slope, and constant of the Cyan-shank partridge chickens, indicating these chickens might maintain their body core temperature via the excellent skin heat dissipation and respiratory heat dissipation capabilities. However, further research is needed to confirm this. As an indicator of heat stress, the Temperature–Humidity Index (THI) is directly proportional to ambient temperature and humidity. This research observed a continuous increase in core body temperature under conditions of elevated temperature and RH, indicating a high THI might place severe heat stress on the chicken.

## 5. Conclusions

Cyan-shank partridge chickens exhibited an elevated core temperature when exposed to a continuous increase in ambient temperature. Segmental regression analysis showed the IPT of the bird core temperature was in the range from 26.52 to 27.02 °C at the ages from 35 to 49 days. The basal core temperature decreased gradually as the bird age increased from 35 to 49 days. Birds can maintain body temperature within a certain range even though the air RH is elevated. It is better to keep the ambient temperature less than 26.5 °C to maintain normal performance of Cyan-shank partridge chickens at the ages from 35 to 49 days during the hot humid summer season. More accurate parameters require further experimental verification. These findings provide a data basis for attenuating heat stress-induced low efficiency of production and energy waste in Cyan-shank partridge chickens.

## Figures and Tables

**Table 1:** Effect of age and RH on the parameters in the BLM of bird core temperature.

Item		(IPT)/°C	Z	Constant/°C
RH	50%	26.52 ± 1.69	0.11 ± 0.04	41.01 ± 0.30
	65%	27.02 ± 1.83	0.11 ± 0.04	41.07 ± 0.29
	80%	26.71 ± 1.81	0.11 ± 0.03	41.02 ± 0.32
Age	35 days	26.64 ± 1.72	0.13 ± 0.03 ^a^	41.33 ± 0.20 ^a^
	42 days	26.93 ± 1.88	0.10 ± 0.03 ^b^	40.96 ± 0.20 ^b^
	49 days	26.68 ± 1.77	0.10 ± 0.04 ^b^	40.80 ± 0.19 ^c^
*p*-value				
RH		0.560	0.672	0.430
Age		0.801	<0.001	<0.001
RH × Age		0.659	0.150	0.728

Different letter superscripts indicate values within same row that are significantly different (*p* < 0.05). *n* = 10. IPT = inflection point temperature; Z = the slope (the change in core temperature with respect to the change in ambient temperature); C = constant (basal core temperature).

## Data Availability

The data supporting the findings of this study are included within the article. Any additional data that support the study’s conclusions can be made available by the corresponding author upon reasonable request.

## Supplementary Materials

The following supporting information can be downloaded at: https://www.mdpi.com/article/10.3390/ani15060820/s1, Table S1: Composition and nutrient level of the basal diet (air-dry basis); Table S2: The means of core temperature of birds as exposed to continuously increased ambient temperature at RH levels of 50%, 65% and 80%; Table S3: Mean and SD of the parameters in the BLM of core temperature at different age stages of birds exposed to three RH levels.
