# Supplementary material for: Changes in Core Temperature of Cyan-Shank Partridge Chickens Exposed to Continuously Increased Ambient Temperature at Different Relative Humidity Levels
Source: Animals (Basel). 2025 Mar 13;15(6):820. doi: 10.3390/ani15060820 (PMC11939733; doi:10.3390/ani15060820)
Supplement: Supplementary file 1 [file animals-15-00820-s001.zip › animals-3506071-supplementary.pdf]

Table S1. Composition and nutrient level of the basal diet (air-dry basis).

Items	Content (22 to 50 days)
Ingredients (g/kg)	
Corn	62.27
Corn gluten meal	6.00
Soybean meal	23.00
Soybean oil	4.00
Limestone	1.40
Dicalcium phosphate	1.60
DL-Methionine	0.80
L-Lysine	0.35
NaCl	0.30
Premix	1.00
Total	100
Calculated nutrient level	
Metabolizable energy (MJ/kg)	13.19
Crude protein (g/kg)	196
Lysine (g/kg)	10.5
Methionine (g/kg)	4.2
Available phosphorus (g/kg)	3.9
Calcium (g/kg)	9.5
Methionine + cystine (g/kg)	7.6

Note: Premix provided per kilogram of diet: vitamin A (transretinyl acetate), 10,000 IU; vitamin D3 (cholecalciferol), 3000 IU; vitamin E (all-rac-α-tocopherol), 30 IU; menadione, 1.3 mg; thiamin, 2.2 mg; riboflavin, 8 mg; nicotinamide, 40 mg; choline chloride, 600 mg; calcium pantothenate, 10 mg; pyridoxine·HCl, 4 mg; biotin, 0.04 mg; folic acid, 1 mg; vitamin B_{12} (cobalamin), 0.013 mg; Fe (from ferrous sulphate), 80 mg; Cu (from copper sulphate), 8.0 mg; Mn (from manganese sulphate), 110 mg; Zn (from zinc oxide), 60 mg; I (from calcium iodate), 1.1 mg; Se (from sodium selenite), 0.3 mg; The nutrient levels were as fed basis; Values based on analysis of triplicate samples of diets.

Table S2 The means of core temperature of birds as exposed to continuously increased ambient temperature at RH levels of 50%, 65% and 80%[a].

Item		RH[b]	35 days						42 days						49 days					
			24°C	26°C	28°C	30°C	32°C	34°C	24°C	26°C	28°C	30°C	32°C	34°C	24°C	26°C	28°C	30°C	32°C	34°C
50%	Mean	51.65	41.3	41.4	41.5	41.7	42	42.3	41.0	41.0	41.1	41.2	41.3	41.5	40.9	40.9	41.0	41.1	41.4	41.6
	SD	3.59	0.2	0.2	0.2	0.2	0.2	0.3	0.3	0.2	0.2	0.2	0.2	0.2	0.3	0.2	0.3	0.3	0.3	0.2
65%	Mean	66.71	41.4	41.5	41.6	41.8	41.9	42.3	41.1	41.1	41.2	41.2	41.3	41.5	41.0	40.9	40.9	41.0	41.1	41.3
	SD	2.31	0.1	0.1	0.2	0.2	0.1	0.2	0.2	0.1	0.1	0.2	0.2	0.1	0.2	0.2	0.1	0.1	0.1	0.1
80%	Mean	79.17	41.4	41.5	41.6	41.7	41.9	42.3	41.0	41.0	41.1	41.2	41.3	41.5	40.8	40.8	40.9	41.0	41.2	41.5
	SD	1.47	0.1	0.1	0.2	0.1	0.1	0.2	0.2	0.2	0.2	0.2	0.2	0.2	0.2	0.2	0.1	0.1	0.1	0.1

Note: [a], means of core temperature of birds at each measured time point (n = 10); [b], means of the measured RH in artificial climate chamber at different relative humidity levels. During the 6-h heating treatment, the relative humidity in the chamber was recorded once every minute and all of recorded values were used to calculate the mean and SD.

Table S3 Mean and SD of the parameters in the BLM of core temperature at different age stages of birds exposed to three RH levels.

**Item**		**(IPT) / °C**	**Z**	**Constant/ °C**
	35 days	26.51 ± 1.60	0.13 ± 0.04	41.30 ± 0.25
50%	42 days	26.20 ± 1.57	0.09 ± 0.04	40.90 ± 0.20
	49 days	26.86 ± 1.99	0.10 ± 0.03	40.82 ± 0.20
	35 days	27.02 ± 1.75	0.13 ± 0.03	41.35 ± 0.17
65%	42 days	27.24 ± 2.28	0.10 ± 0.02	41.05 ± 0.19
	49 days	26.81 ± 1.57	0.09 ± 0.04	40.81 ± 0.20
	35 days	26.39 ± 1.90	0.12 ± 0.03	41.35 ± 0.21
80%	42 days	27.35 ± 1.67	0.10 ± 0.02	40.94 ± 0.20
	49 days	26.38 ± 1.87	0.12 ± 0.03	40.76 ± 0.19

[1] Different letter superscripts indicate values within same row that are significantly different ($p < 0.05$). n = 10. IPT = inflection point temperature; Z = the slop (the change in core temperature with respect to the change in ambient temperature); C = constant (basal core temperature).
